# Complication avoidance, rehabilitation, pain therapy and palliative care for patients with metastatic spine tumors: WFNS spine committee recommendations

**DOI:** 10.1007/s10143-024-03050-3

**Published:** 2024-10-30

**Authors:** Mirza Pojskic, Sait Naderi, Sandeep Vaishya, Mehmet Zileli, Francesco Costa, Salman Sharif, Ziya L. Gokaslan

**Affiliations:** 1 Department of Neurosurgery, University of Marburg, Marburg, Germany; 2 Department of Neurosurgery, Istanbul Brain and Spine Center, Istanbul, Türkiye Turkey; 3 Department of Neurosurgery, Fortis Memorial Research Institute, Guragaon and Fortis Hospital Vasant Kunj, New Delhi, India; 4 Fortis Memorial Hospital, New Delhi, India; 5 Department of Neurosurgery, Sanko University, Gaziantep, Türkiye Turkey; 6 Spine Surgery Unit, Department of Neurosurgery, Fondazione IRCCS Istituto Neurologico Carlo Besta, Milan, Italy; 7 Department of Neurosurgery, Liaquat National Hospital and Medical College, Karachi, Pakistan; 8 Department of Neurosurgery, The Warren Alpert Medical School of Brown University, Providence, RI USA

**Keywords:** Spine metastases, Complications, Pain therapy, Palliative care

## Abstract

Objective: This review aims to formulate the most current, evidence-based recommendations regarding complication avoidance, rehabilitation, pain therapy and palliative care for patients with metastatic spine tumors. Methods: A systematic literature search in PubMed and MEDLINE, and was performed from 2013 to 2023 using the search terms “complications” + “spine metastases”, “spine metastases” + + “rehabilitation”, “spine metastases” + “pain therapy” + “palliative care”. Screening criteria resulted in 35, 15 and 56 studies respectively that were analyzed. Using the Delphi method and two rounds of voting at two separate international meetings, nine members of the WFNS (World Federation of Neurosurgical Societies) Spine Committee generated nine final consensus statements. Results: Preoperative assessment for complications following surgery in patients with metastatic spine tumors should include estimation of Karnofsky score, site of primary tumor, number of spinal and visceral metastasis, ASA score and preoperative Hb (Hemoglobin) value. Complication risk factors are age > 65 years, preoperative ASA score of 3 and 4 and greater operative blood loss. Pain management using WHO analgesic concept and early mobilization are needed, starting with non-opioids, weak opioids followed by strong opioids. Morphine is the first choice for moderate to severe pain whereas IV-PCA may be used for severe breakthrough pain with monitoring. Use of bisphosphonates is considered in cases of non-localized pain and not accessible radiation therapy. Conclusions: These nine final consensus statements provide current, evidence-based guidelines on complication avoidance, rehabilitation, pain therapy and palliative care for patients with spinal metastases.

**Supplementary Information:**

The online version contains supplementary material available at 10.1007/s10143-024-03050-3.

## Introduction

Spinal metastases, predominantly from lung, prostate and breast cancer, have an incidence of 15.67%. Metastatic epidural spinal cord compression (MESCC) occurs in almost 10% of these patients, with pathological fracture in 1 out of 8 patients [1]. In up to 1/5 of all cases, neurological deficits and pain with spinal cord compression which warrant surgical therapy occur [2]. Surgical complication rate is estimated between 10% and 66.7% [3, 4]. Since there is an increase in total number of surgeries for spinal metastases followed by improvement of technique, increase of complications is expected [3]. Recovery due to deficits caused by MESCC is facilitated also through rehabilitation, similarly as in cases of traumatic spinal injury [5, 6]. Palliative therapy and pain management are important in preservation of patient Quality of Life (QoL) even in the latter stage cancer course [7]. Pain of moderate to severe intensity occurs in more than 50% of all cancer patients [8].

The goal of this work is to produce the latest evidence-based recommendations on avoidance of complications in treatment of patients with spinal metastases, on role of rehabilitation, pain therapy and palliative care, with a particular relevance for practicing spinal surgeons in low-and middle income countries. The goals of this specific paper are to summarize the latest evidence on the preoperative and intraoperative risk factors for complications, strategies on avoidance of complications and postoperative monitoring, current concepts of mobilization and rehabilitation of these patients and to analyze guidelines and current literature on pain therapy and palliative care. The World Federation of Neurosurgical Societies (WFNS) Spine Committee formulated nine final consensus statements on LHD via two-rounds of Delphi meetings.

## Materials and methods

The systematic review and meta-analysis were conducted following PRISMA (Preferred Reporting Items for Systematic Reviews and Meta-Analyses) and Cochrane guidelines [9] .

### Search strategy

A systematic literature search in PubMed, MEDLINE, and CENTRAL was performed from 2013 to 2023 using the search terms “complications” + “spine metastases”, “spine metastases” + “rehabilitation”, “spine metastases” + “pain therapy” + “palliative care”. Only articles that specifically dealt with aspects of complications, rehabilitation, pain therapy and palliative care in the context of spinal metastases were taken into consideration. We focused explicitly on official guidelines of neurosurgical and spine societies, randomized controlled trials, and retrospective and prospective studies with more than 50 patients. Case reports with less than 50 patients, nonhuman studies, studies without full text available, and studies not in English were excluded.

In addition list of eligible trials and reviews was manually checked by coauthors. A complete search strategy is available. The coauthors screened titles and abstracts of all records after duplicates were removed, followed by screening of full texts. All authors have used a standardized data extraction form to collate study characteristics (publication year, country, number of patients), and main subject of the study (complications, rehabilitation, pain therapy and palliative care).

**Fig. 1:**
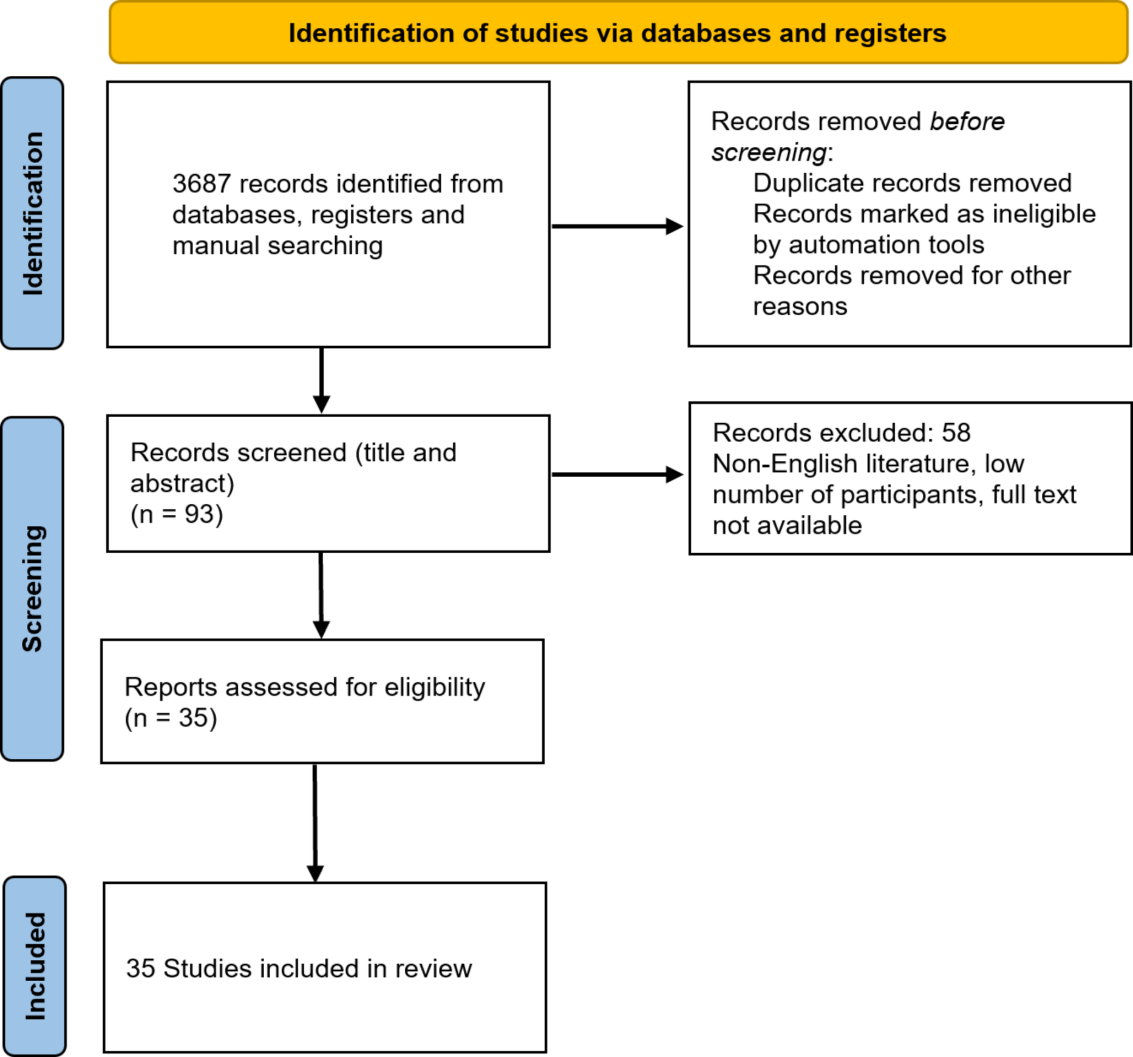
PRISMA flow chart for identification of studies for complications and complications avoidance in spine metastases

**Fig. 2:**
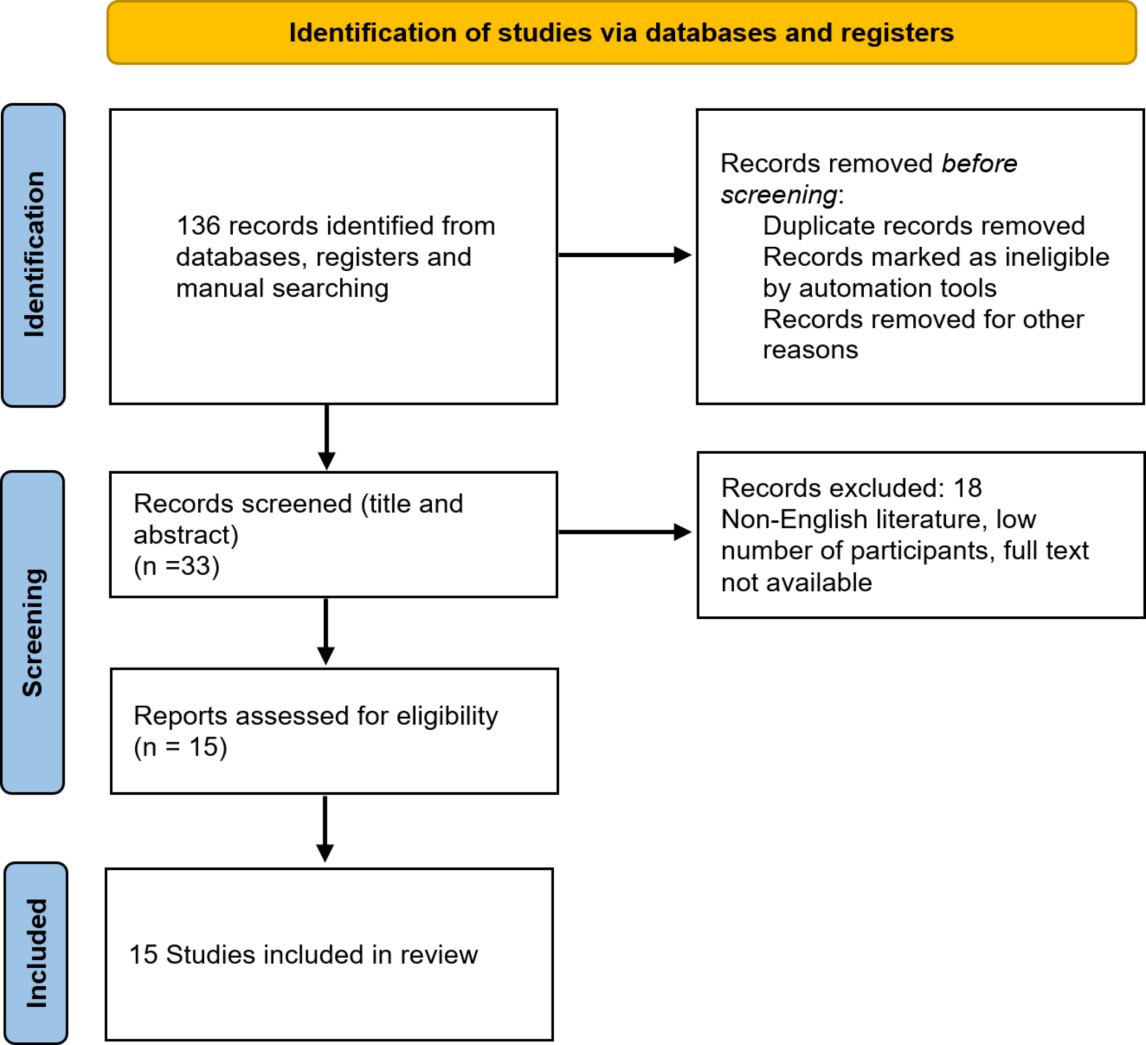
PRISMA flow chart for rehabilitation in spine metastases

**Fig. 3:**
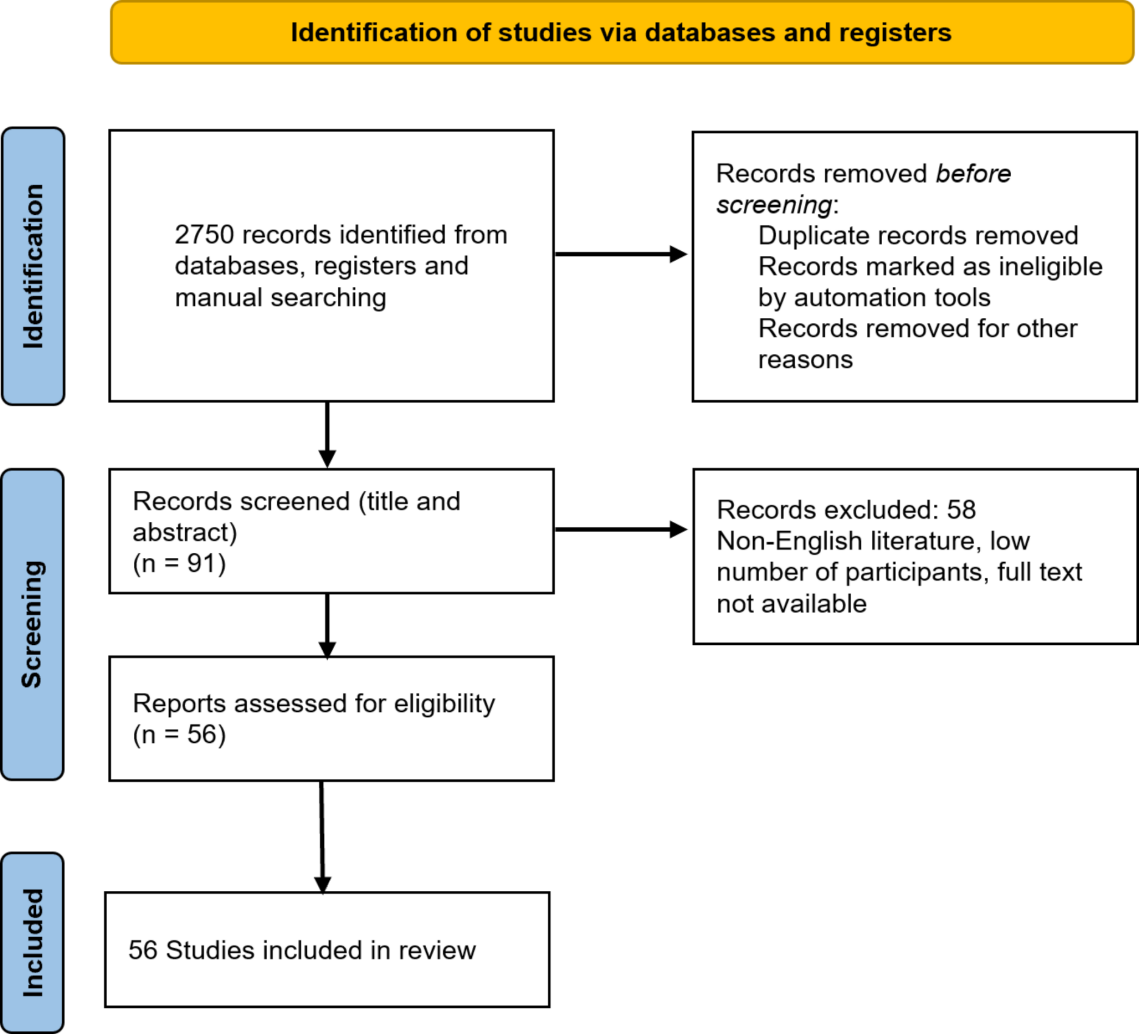
PRISMA flow chart for pain therapy and palliative care in patients with spine metastases

For complications and complication avoidance of spine metastases, 3687 articles across all databases were obtained. After removing duplicates, abstract review by two independent reviewers, and full text review of the remaining studies, the authors selected 35 studies for analysis (Fig. 1). For rehabilitation of patients with spine metastases, articles were obtained. After removing duplicates, abstract, and full text review, 15 studies were included in the final analysis (Fig. 2). For pain therapy and palliative care, 2750 articles were initially obtained. Full text review of manuscripts was performed, resulting in total 56 studies included in the final analysis (Fig. 3).

### Consensus meetings

An international committee of spinal surgeons, specifically members of the World Federation of Neurosurgical Societies (WFNS) Spine Committee, organized two consensus meetings on spinal metastases, the first of which was conducted in Karad, India, in January 2023, and the second in Belgrade, Serbia, in October 2023. Set of statements on spinal metastases was provided by each participant and discussed at the initial meeting. After a preliminary voting session, some statements were excluded because of the low evidence of existing literature. Nine revised statements were voted on at the second meeting.

We utilized the Delphi method to generate our consensus statements. The level of agreement or disagreement on each item was voted independently in a blind fashion through a Likert-type scale from 1 to 5 (1 = strongly agree, 2 = agree, 3 = somewhat agree, 4 = disagree, 5 = strongly disagree). Results were presented as a percentage of respondents who scored each item as 1, 2, or 3 (agreement) or as 4 or 5 (disagreement). When agreement or disagreement sum was achieved > 66%, consensus was declared. See Table 1 that shows final voting on the final nine statements.

**Table 1:** Final voting results on nine statements

Statement	No of respondents of agreement according to Likert scale/Percent
1- Preoperative assessment for complications following surgery in patients with metastatic spine tumors should include estimation of Karnofsky score, primary tumor, number of spinal and visceral metastasis, ASA score and preoperative Hb (Hemoglobin) value	9/9 agreement 100%
2- There is evidence that advanced age (> 65 years) is a significant risk factor for complications in treatment of patients with spinal metastases. (9/9 agreement 100%)	9 /9 agreement 100%
3- Preoperative ASA score of 3 and 4 increases risk of medical and surgical complications in patients with spinal metastasis. Surgical treatment planning in these patients needs additional consideration in respect of duration and invasiveness of surgery and estimated blood loss.	5/8 agreement 87.5%
4- Patients who underwent surgery for spine metastasis with greater blood loss have increased likelihood for cardiovascular events, pulmonary insufficiency and cerebral events, therefore these patients need careful monitoring of these potential postoperative complications.	9/9 agreement 100%
5- Early postoperative mobilization and multimodal pain management are encouraged for high-risk patients with spinal metastasis	9/9 agreement 100%
6- Pain management and palliation in spinal metastasis should be performed as indicated by the WHO analgesic ladder appropriate for the severity of pain starting with non-opioids, weak opioids followed by strong opioids	8/8 agreement 100%
7- The opioid of first choice for moderate to severe cancer pain is oral morphine [1, A], with a different opioid to be considered in the absence of adequate pain control (despite opioid dose escalation) or in the presence of unacceptable opioid side effects	8/8 agreement 100%
8- IV-PCA may provide timely, safe, and useful analgesia for patient with severe breakthrough pain, however clinicians need to closely monitor delirium and toxicity in advanced cancer patients	8/8 agreement 100%
9- Bisphosphonates may be considered for the treatment of patients with bone metastases with a good prognosis, especially when pain is not localized or RT is not readily accessible	8/8 agreement 100%

## Results and discussion

### Complications and avoidance of complications in spine metastases

Surgical complications can be divided in strictly surgical complications, complications regarding anesthesia and patient positioning as well as [3]. 6.5% postoperative surgical site infection (SSI) rate with 8.3% re-operation rate was reported as the main complication following surgery for spinal metastases, followed by postoperative neurological deterioration 3.3% and hardware failure 2% in a recent literature review [10]. Choi et al. [11, 12] performed a prospective study on 1430 patients with spinal metastases to analyze surgery outcomes. Presence of spinal and visceral metastases, tumor type as well as Karnofsky score were identified as important for prognosis in respect to surgical treatment [11]. Karnofsky score estimates preoperative general condition and in combination with tumor histology and staging expressed through number of spinal and other distant metastases deems proper selection of candidates for surgery. Further important factors are ASA score and preoperative Hemoglobin (Hb) value [11, 13]. Complication risk assessment includes also choice of surgical technique– decompression, decompression + fusion as well as open vs. percutaneous stabilization, due to differences in wound surface and blood loss. There are several strategies to prevent postoperative infection: local antibiotics, wound surface minimization through employment of minimally invasive techniques, timing of adjuvant radiotherapy and chemotherapy following surgery. Several factors which were identified to increase morbidity following surgery are age of 40 and older, with further significant increase in older than 65 years, surgery on three levels and more, surgery on patients who had previously radiotherapy as well as myelopathy presence and surgeons experience [14]. Estimation of complications risk can be performed indirectly using traditional scoring systems such as Tokuhashi [15] or Spinal Metastasis Invasiveness Index (SMII) [16], which predicts high blood loss and prolonged duration of surgery. One further prognostic score, New England Spinal Metastasis Score (NESMS), which was created using NSQIP (National Surgical Quality Improvement Program) database with 776 patients [17, 18] reported a 30-day mortality rate of 11% (*N* = 87), and morbidity including one or more complications in 51% of patients (*N* = 395). NESMS score of 3 has shown 74% reduction in major systemic complications and an 88% reduction in failure to rescue [18].

Surgeons should not be biased against operating elderly patients, as prospective multicenter study by the Global Spine Tumor Study concluded [19]. Patients older than 80 years show less neurological improvement and worse survival rate compared to younger ones, which might be due to the fact that these patients, although sometimes with good ASA status and fair functional status, more often undergo emergency and palliative procedures. Operating the elderly is compounded by the fact that they undergo more emergency and palliative procedures, despite good ASA scores and functional status [19]. Yonezawa et al. report on 129 perioperative complications in 76 of 112 surgeries in their cohort, with increased complication rate in elderly patients; however, prognosis and local control shows improvement following resection, especially in cases of primary tumors such as renal cell and thyroid cancer [20]. Multicenter surveillance study from German Spinal Registry (DWG-Register) [21] which included 1617 decompression surgeries with and without instrumentation identified that the overall prevalence of a major postoperative complication for patients with spinal metastases was 16.5%, and prevalence of intraoperative complications 8%. The likelihood ratio for major complications by blood loss greater than 500 mL were as follows: cardiovascular event 4.22, pulmonary insufficiency 4.18, and cerebral event 5.47 [21]. Higher risk of complications is independently predicted by preoperative status with ASA score of 3 and 4, invasiveness or surgery, blood loss > 500 mL and necessity and quantity of blood transfusions. Further study from the same registry on 528 patients identified obesity as a risk factor, since these patients were predisposed to have blood loss more than 500 mL more often than nonobese patients, and were more likely to have ASA score of 3 and 4 [22].

There were several studies on wound healing deficits in patients with spinal metastases. Study of Keam et al. [23] on 165 patients who underwent surgical treatment and had prior radiation therapy, did not find a correlation between radiation and extensive wound healing problems. An analysis of 205 patients from Varga et al. [24] revealed that there were no differences in incidence of wound healing deficits and reoperations regardless of preoperative or postoperative radiotherapy or no radiotherapy at all. An optimal radiation therapy-surgery interval has not been defined yet, however based on published literature and expert opinions, an interval of 2 weeks, the minimum being 7 days, is recommended, with possibility of reduction in postop-stereotactic body RT [25]. Further conclusions were that if RT-surgery window is > 12 months, wound-complications rise, and that postop-RT has fewer wound complications versus preop-RT [25]. There are no studies which are specific for spine metastases and prevention of wound healing deficits using topical antibiotics. The data from the literature are somewhat contradictory – a randomized controlled trial has shown that intra-wound vancomycin has no effect on SSI; in addition it has shown to increase the rate of gram-negative infections [26], whereas systematic review by Zhou et al. [27]. demonstrated lower incidence of SSI with local application of vancomycin powder ( 1.9% vs. 4.8%). Zhou et al. have also investigated differences in invasiveness of surgery in relation to incidence of wound healing deficits and found that highest incidence as in surgery with instrumentation, in open surgery and in patients with neuromuscular diseases [27]. Similarly, highest wound revision rate was found following instrumentation in analysis of Han-Dong Lee et al., with lowest rate for vertebroplasty and decompression only [28].

Adjuvant minimaly invasive interventions have emerged in cases of frail patients who are not suitable candidates for surgery as well as in patients with mild instability of the spine, such as ablation techniques which use intraoperative MRI guidance to place a probe within the target lesion and to monitor temperature-dependent killing of the tumor cells [29]. Promising strategies which have emerged as less invasive alternatives in treatment of spinal metastases are a combination of radiofrequency ablation (RFA) and vertebral augmentation, such as balloon kyphoplasty and other techniques [30]. Recent systematic review which included 947 patients from 25 studies who underwent this combined treatment revealed that significant pain reduction was noted, with a low complication rate of 1% [30]. Most common complications were radiculopathy, which was usually not permanent, as well as extravasation of cement, which was asymptomatic [30]. Implementation of these therapy modalities has also shown favorable effects on local tumor progression control, especially in cases of lesions located within the vertebral body compared to those with involvement of posterior elements, with a rate of only 5% in short-term and long-term follow-up, and rate of 22% in mid-term follow up [30] and with consistently low tumor progression rates throughout the literature. This review noted a very high local tumor control of 91% [30]. Other systematic analyses suggested that microwave ablation (MWA) in combination with surgery might be more beneficial in terms of local tumor control compared to RFA, however with a significantly higher complication rate of MWA compared to RFA (27.4 vs. 10.9%) [31].

The role of types of surgery on complication rate was also investigated. An analysis of National Readmission Database on 4423 patients has shown that spinal fusion and combined fusion and decompression were less likely to have a 30-day readmission compared to spinal decompression alone (difference in comparison to lumbar degenerative spine surgery) [32]. Postoperative infection, acute post-hemorrhagic anemia and genitourinary complication with sepsis were the most common reason for readmission [32]. Contrary to this finding, Lenschow et al. [33] in their analysis of 301 patients reported that complications occurred more often in instrumented than non-instrumented patients, without differences in neurological outcome. Invasiveness of surgery has also shown correlation to incidence of complications, as shown by Pranata et al. in systematic review of 8 studies and 486 patients, where MISS was associated with lower complications, lower blood loss and transfusion rate, and shorter length of stay without effect on neurological outcome and operative time [34]. When comparing laminectomy to corpectomy [35], a greater 30-day postoperative complication rate among patients undergoing corpectomy was noted.

Recently, concept of readmission-free survival was defined as the time duration between discharge after index-operation and first unplanned hospital readmission (UHR)/ death [36]. Short-term readmission free survival, i.e. under 90 days, was found to be influenced by preoperative hemoglobin (Hb) level > 12 g/dL, ≤ 3 comorbidities, shorter index length of stay ≤ 10 days and absence of neurologic/hematologic complications during index stay, whereas Hb > 12 g/dL as well as primary tumors with advanced treatment modalities were shown to influence readmission after 90 days [36]. 30 days mortality has shown correlation to steroid use, transfusions, infections, smoking and presence of bleeding disorders [37]. 

### Rehabilitation of patients with spinal metastases

Main goals of rehabilitation following surgery for MESCC are optimization of the remaining neurological function to increase patient autonomy and preserve quality of life, as well as providing for assistive devices to improve patient autonomy and safety [6]. An analysis of 309 patients with spine metastases, of which 177 were included into in “Enhanced Recovery After Surgery” (ERAS) program and comparison to non-EAS group has shown that ERAS cohort had decreased estimated blood loss, mean opioid use in the first five days following surgery, earlier removal of urinary catheter and earlier ambulation [38, 39]. ERAS program has led to reduced hospital stay as well as reduced opioid use [38]. In UK, a six weeks inpatient Specialist Spinal Rehabilitation program was initiated for patients with MESCC [40], with improvement in the Spinal Cord Independence Measure, independent of age. Prior to rehabilitation it is necessary to rule out fractures, since rehabilitation for patients with bone metastases increases the risk of adverse events, including pathological fractures and paralysis. Therefore, risk assessment for fractures prior to rehabilitation, for example using SINS index, is necessary [41].

### Pain therapy and palliative care for patients with spinal metastases

Physical, emotional and spiritual distress in relation to neurological complications and distress should be treated with palliative care [6]. Patient expectation as well as prognosis should be taken into account in terms of palliative care, as recommended by The European Association of Palliative Care [42]. The WHO (2018) defines palliative care as an “approach that improves the quality of life of patients and their families facing the problems associated with life-threatening illness, through the prevention and relief of suffering by means of early identification and impeccable assessment and treatment of pain and other problems, physical, psychosocial and spiritual” [43]. 

Oncological pain therapy is defined by analgesic ladder proposed by WHO [44], yet without standardization of cancer pain classification [8]. Need for opioid therapy due to chronic pain is present in up to 50% of patients at the beginning of the cancer disease, and progresses in 75–90% in advanced stage [45]. There is a trend of replacement of invasive procedures (en-bloc spondylectomies) by less invasive separation surgery, then the use of MISS due to lower complication rate, and stereotactic radiosurgery [46]. Furthermore, in the era of targeted therapies which change treatment of oncological patients, modern treatment principles are based on decision made by multidisciplinary team with oncologists, radiation therapists, surgeons, interventionalists, and pain specialists is required [46].Pain management includes variety of different modalities such as analgesics, blocks, PCA, radio ablation, combination of RFA and other treatments, augmentation procedures and spinal cord stimulation. ESMO clinical guidelines defined in 2012 that “pain management and palliation in spinal metastases should be performed step by step as indicated by the WHO analgesic ladder appropriate for the severity of pain starting with Non opioids, Weak opioids followed by Strong opioids. Adjuvant can be added to pain therapy in all steps to increase their effectiveness with PCA as an important option. [II, B]“ [47]. Pain intensity and outcome of treatment according to ESMO guidelines as well as EAPC evidence-based warrants assessment using VAS scale as well as with assessment of psychosocial distress [48–51]. 

Palliative radiotherapy enables in 60% of patients sufficient pain relief with age, numerical rating scale (NRS), and biological effective dose (BED10) as important factors which influence pain response in spinal metastases [52]. Nakata et al. [53] investigated pain response to radiotherapy in 109 patients with spine metastases without paralysis and reported that pain disappeared in 88% of the patients with spinal stability (SINS < 7) and in 58% of the patients with spinal instability (SINS ≥ 7). In all patients with bone metastases who experience pain, external beam radiotherapy (EBRT) was proposed with a single dose of 8 Gy and possibility of re-radiation in cases of recurrent pain [54]. Radiofrequency ablation (RFA) with or without cement injection in cases of osteolytic bone metastases has shown pain relief in prospective series with 34 patients [55]. When it comes to osteolytic lesions, mechanical stability can be provided with cement and RFA [56]. Giammalva et al. [57] demonstrated a significant pain reduction in 54 patients with thoraco-lumbar metastatic vertebral fractures with combination treatment of vertebroplasty, RFA and transpedicular screw fixation. Vertebral augmentation with RFA [58] is also called augmentation surgery, and it has shown that it leads to relief in patients with severe intractable pain and stable metastatic compression vertebral fractures. Further techniques include microwave ablation with osteoplasty [59] which has shown pain reduction in patients with persistent or recurrent pain after radiation therapy, patients who were not candidates or declined radiotherapy [60]. Percutaneous image-guided cryoablation is further method for pain relief and achieving local control [61].

According to ECMO guidelines, for mild to moderate pain, weak opioids such as tramadol, dihydrocodeine and codeine can be given in combination with non-opioid analgesics. As an alternative to weak opioids, although not a part of WHO scheme, low doses of strong opioids could be an option [49]. Paracetamol and/or a NSAID are effective in short term for treating all intensities of pain [62]. ESMO [49, 50] guidelines further define oral morphine as the first choice opioid for moderate to severe pain. First-choice alternative route for patients unable to receive opioids by oral is s.c. route (morphine, diamorphine and hydromorphone) [49, 50]. I.v. infusion should be considered when s.c. administration is contraindicated, such as in cases of peripheral oedema, coagulation disorders, poor peripheral circulation and need for high volumes and doses [49, 50]. 

Targeted therapies for bone metastatic pain include bisphosphonates and denosumab [63]. Bisphosphonates and denosumab are thought to delay the onset of pain [64], whereas bisphosphonates are not used for localized pain [65–67]. Alternative to bisphosphonates is denosumab, especially for delaying bone pain recurrence in patients with metastases of solid tumors and multiple myeloma [68–70]. Systematic review within the European Association for Palliative Care guidelines project revealed that there is no sufficient evidence to support use of this medication for pain treatment [64].

Neuropathic cancer pain (NCP) is caused by nerve damage attributable to the cancer, and/or oncological treatments such as chemotherapy, radiotherapy, and surgery, with prevalence up to 40% [71, 72]. Tricyclic antidepressants (TCAs) are used for treatment together with serotonin and norepinephrine reuptake inhibitors (SNRIs) [73]. Recommendation for NCP treatment as monotherapy exists for gabapentin, pregabalin, duloxetine and TCA (doses ≤ 75 mg/day) with, n case of insufficient analgesia, an opioid switching concept, which contains use of adjuvant drugs such as gabapentinoids,, and antidepressants (amitriptyline) as well as methadone, although titration and dose conversion need to be taken into consideration [73]. Novel agents include Tetrodotoxin, Botulinum Toxin Type A (BoNT-A), TRPM8 Activator Menthol, Growth Factors Inhibitors and Lemairamin [73].

For severe, breakthrough pain, intravenous patient-controlled analgesia (IV-PCA) can provide sufficient pain relief and can help titration of opioids, weaning to oral analgesia and to decide for interventional procedures [74]. Monitoring is warranted for side effects, such as risk of delirium [75]. Recent database analysis on over 11,000 patients revealed that intravenous PCA use after surgery ( continuous or total volume of infusions), was significantly associated with the occurrence and severity of postoperative pain both in the first and second 24 h postoperatively, which suggest that the pain control was not sufficient [76]. Finaly, spinal cord stimulation was described in smaller case series as useful for treatment of refractory cancer pain and chemotherapy-related pain [77]. Intrathecal morphine pump can also be considered for refractory cancer pain.

## WFNS Spine committee recommendations

After summarizing and discussing the available literature, as outlined above, the WFNS achieved consensus on the following nine statements.

Complication avoidance and rehabilitation of geriatric patients with metastatic vertebral tumors:

### 1

Preoperative assessment for complications following surgery in patients with metastatic spine tumors should include estimation of Karnofsky score, primary tumor, number of spinal and visceral metastasis, ASA score and preoperative Hb (Hemoglobin) value. (9/9 agreement 100%)

### 2

There is evidence that advanced age (> 65 years) is a significant risk factor for complications in treatment of patients with spinal metastases. (9/9 agreement 100%)

### 3

Preoperative ASA score of 3 and 4 increases risk of medical and surgical complications in patients with spinal metastasis. Surgical treatment planning in these patients needs additional consideration in respect of duration and invasiveness of surgery and estimated blood loss. (5/8 agreement 87.5%)

### 4

Patients who underwent surgery for spine metastasis with greater blood loss have increased likelihood for cardiovascular events, pulmonary insufficiency, and cerebral events, therefore these patients need careful monitoring of these potential postoperative complications. (9/9 agreement 100%)

### 5

Early postoperative mobilization and multimodal pain management are encouraged for high-risk patients with spinal metastasis. (9/9 agreement 100%)

Pain therapy and palliative care for metastatic vertebral tumors:

### 6

Pain management and palliation in spinal metastasis should be performed as indicated by the WHO analgesic ladder appropriate for the severity of pain starting with non-opioids, weak opioids followed by strong opioids. [II, B] (8/8 agreement 100%).

### 7

The opioid of first choice for moderate to severe cancer pain is oral morphine [1, A], with a different opioid to be considered in the absence of adequate pain control (despite opioid dose escalation) or in the presence of unacceptable opioid side effects. [III, C] (8/8 agreement 100%).

### 8

IV-PCA may provide timely, safe, and useful analgesia for patient with severe breakthrough pain, however clinicians need to closely monitor delirium and toxicity in advanced cancer patients. (3 C) (8/8 agreement 100%).

### 9

Bi sphosphonates may be considered for the treatment of patients with bone metastases with a good prognosis, especially when pain is not localized or RT is not readily accessible [2, C]. (8/8 agreement 100%)

## Conclusion

These nine final consensus statements provide current, evidence-based guidelines on the complication avoidance, rehabilitation, pain therapy and palliative care for patients with spinal metastases. Preoperative assessment for complications following surgery is obligatory and must include assessment of general condition of the patient, site of primary tumor, number of spinal and visceral metastasis, ASA score and preoperative Hb value. Advanced age > 65 years as well as preoperative ASA score of 3 and 4 are risk factors for complications and special care needs to be taken in such patients. Surgery with greater blood loss increases likelihood for cardiovascular and cerebral events and pulmonary insufficiency, so these patients need careful postoperative monitoring. Multimodal pain management according to WHO analgesic ladder and early mobilization are encouraged. For moderate to severe cancer pain the first-choice opioid must be oral morphine. Use of IV-PCA for severe pain can be useful, however close monitoring of delirium and toxicity is mandatory. In patients with good prognosis, non-localized pain and no availability of radiation therapy, bisphosphonates may be considered for the treatment.

## Electronic supplementary material

Below is the link to the electronic supplementary material.

Supplementary Material 1

## Data Availability

No datasets were generated or analysed during the current study.

## Abbreviations

ASA: American Society of Anesthesiologists
BED: Biological effective dose
CT: Computed tomography
EAPC: The European Association of Palliative Care
EBRT: External Beam Radiation Therapy
ERAS: Enhanced recovery after surgery
ESMO: European Society for Medical Oncology
Hb: Hemoglobin
IV PCA: Intravenous patient controlled analgesia
MESCC: Metastatic epidural spinal cord compression
MISS: Minimally invasive spine surgery
MRI: Magnetic resonance imaging
NCP: Neuropathic cancer pain
NESMS: New England Spinal Metastasis Score
NRS: Numerical rating scale
NSAID: Non-steroidal anti-inflammatory drug
NSQIP: National Surgical Quality Improvement Program
RFA: Radiofrequency ablation
RFS: Readmission free survival
RT: Radiation therapy
SINS: Spinal Instability Neoplastic Score
SM: Spinal metastases
SMII: Spinal Metastasis Invasiveness Index
SNRI: Serotonin and norepinephrine reuptake inhibitors
SSI: Surgical site infection
TCA: Tricyclic antidepressants
UHR: Unplanned hospital readmission
VAS: Visual analogue scale
VCF: Vertebral compression fracture
WFNS: World Federation of Neurosurgical Societies
WHO: World Health Organization
