# Supplementary material for: Complication avoidance, rehabilitation, pain therapy and palliative care for patients with metastatic spine tumors: WFNS spine committee recommendations
Source: Neurosurg Rev. 2024 Oct 30;47(1):830. doi: 10.1007/s10143-024-03050-3 (PMC11525440; doi:10.1007/s10143-024-03050-3)
Supplement: Supplementary file 1 — Supplementary Material 1 [file 10143_2024_3050_MOESM1_ESM.pdf]

PD Dr.med. Mirza Pojskic

Department of Neurosurgery

University Hospital Marburg

Philipps University Marburg

Marburg, Germany

Marburg, 03.10.2024

Cover letter

Editor-in-Chief

Daniel M. Prevedello, MD, MBA, Department of Neurological Surgery, The Ohio State University, USA

Prof. Dr. med. Joachim Oertel, MD, PhD

Editor

Klinik für Neurochirurgie, Universitätsklinikum des Saarlandes, Germany

Dear Editors,

I am pleased to submit our revised manuscript entitled " Complication avoidance, rehabilitation, pain therapy and palliative care for patients with metastatic spine tumors: WFNS Spine Committee Recommendations" by Mirza Pojskić , Sait Naderi, Sandeep Vaishya, Mehmet Zileli, Francesco Costa, Salman Sharif and Ziya Gokaslan to be considered for publication in your esteemed journal. Followed by evaluation of the two esteemed reviewers and initial positive feedback, here we provide the revised and improved version of our manuscript.

The World Federation of Neurosurgical Societies (WFNS) Spine Committee initiated efforts to formulate recommendations regarding the epidemiology and prevention, clinical and neuroradiological diagnosis, conservative and surgical treatment, complications, pain therapy as well as rehabilitation of various spinal diseases through the published evidence and using elaborated methodology, with formulation of recommendations to improve patient care by defining the relevant literature and decision-making processes involved.

Recommendations are aimed at practicing neurosurgeons and spine surgeons in the entire world. Several guidelines on treatment of metastatic spine tumors already exist, however we aimed to provide an update combining these with current knowledge and statement recommendations based on analysis of recently published current literature.

I, Mirza Pojskic, certify that this manuscript is a unique submission and is not being considered for publication, in part or in full, with any other source in any medium. We hope that our manuscript to be carefully read and found suitable for publication in the journal "Neurosurgical Review".

Sincerely,

Mirza Pojskić, MD, Assistant Professor

Department of Neurosurgery, University Marburg, Germany

Here is our point-to-point response to reviewers:

Reviewer 1

Point 1. Very nice review and discussion. There is evidence that RFA of spine mets helps with preventing spread and improves XRT success. You may want to add this information into your discussion and cite some papers.

Response 1. We thank Reviewer 1 for careful evaluation of our study, positive comment and suggestion for improvement. We have added the following paragraph on role of RFA in treatment of spine metastases in the Discussion:

Adjuvant minimaly invasive interventions have emerged in cases of frail patients who are not suitable candidates for surgery as well as in patients with mild instability of the spine, such as ablation techniques which use intraoperative MRI guidance to place a probe within the target lesion and to monitor temperature-dependent killing of the tumor cells[29]. Promising strategies which have emerged as less invasive alternatives in treatment of spinal metastases are a combination of radiofrequency ablation (RFA) and vertebral augmentation[30]. Recent systematic review which included 947 patients from 25 studies who underwent this combined treatment revealed that significant pain reduction was noted, with a low complication rate of 1%[30]. Most common complications were radiculopathy, which

was usually not permanent, as well as extravasation of cement, which was asymptomatic[30]. Implementation of these therapy modalities has also shown favorable effects on local tumor progression control, especially in cases of lesions located within the vertebral body compared to those with involvement of posterior elements, with a rate of only 5% in short-term and long-term follow-up, and rate of 22% in mid-term follow up[30], with consistently low tumor progression rates throughout the literature. This review noted a very high local tumor control of 91%[30]. Other systematic analyses suggested that microwave ablation (MWA) in combination with surgery might be more beneficial in terms of local tumor control compared to RFA, however with a significantly higher complication rate of MWA compared to RFA (27.4 vs 10.9 %)[31].

Reviewer 2

Point 1. I think that avoiding spinal metastatic tumor complications article will help resolve the confusion in this area. The material method section of the meta-analysis conducted for this purpose is suitable for systematic assignment. It is seen that each of the consensus items reached with the study is supported by the literature. In the discussion, inferences were made on the main topic and presented in detail on an item-by-item basis. are many incorrect punctuations and misstypings in the text (″patients. 1. 1. ″ ″meeting.. ″ ″PCA,, radioablation,, ″). When evaluated from this perspective, I think the article should be accepted after the writing errors are corrected. Minor revision.

Response 1. We thank Reviewer 2 for careful evaluation of our study, positive comment and suggestion for improvement. We have cross-checked the manuscript and corrected the typos.
